# EBV-positive diffuse large B cell lymphoma secondary to activated phosphoinositide 3 kinase δ syndrome type 1 (APDS1): a case report and literature review

**DOI:** 10.3389/fimmu.2025.1583405

**Published:** 2025-05-08

**Authors:** Qiu-yuan Xiang, Min Zuo, Ji-Hao Zhou, Chun Feng

**Affiliations:** ^1^ Department of Hematology, The Second Clinical Medical College of Jinan University, Shenzhen, China; ^2^ Department of Pathology, Shenzhen People’s Hospital (The Second Clinical Medical College of Jinan University; The First Affiliated Hospital of Southern University of Science and Technology), Shenzhen, China; ^3^ Department of Hematology, Shenzhen People’s Hospital (The Second Clinical Medical College of Jinan University; The First Affiliated Hospital of Southern University of Science and Technology), Shenzhen, China

**Keywords:** activated phosphoinositide 3-kinase δ syndrome, Epstein-Barr virus, diffuse large B-cell lymphoma, Epstein-Barr virus-positive diffuse large B-cell lymphoma (EBV+ DLBCL), inborn error of immunity

## Abstract

Activated phosphoinositide 3-kinase δ syndrome (APDS), an inborn error of immunity associated with gain-of-function mutations in the *PIK3CD* gene, is characterized by dysregulated PI3Kδ signaling. The clinical spectrum commonly includes recurrent respiratory infections and lymphoproliferative manifestations. We present an adolescent male with APDS1 manifesting recurrent sinopulmonary infections, generalized lymphadenopathy, hepatosplenomegaly, gastrointestinal manifestations, and combined T-cell/B-cell lymphopenia, complicated by Epstein-Barr virus-positive diffuse large B-cell lymphoma (EBV+ DLBCL). Whole-exome sequencing identified a heterozygous *PIK3CD* variant (c.3061G>A p.Glu1021Lys), supporting the molecular diagnosis of APDS1. This case adds to emerging evidence linking APDS1 with EBV-driven lymphomagenesis, thereby further supporting the critical role of PI3K δ pathway dysregulation in promoting EBV-associated lymphoid malignancies.

## Introduction

Activated phosphoinositide 3-kinase δ syndrome (APDS) is an autosomal dominant primary combined immunodeficiency disorder characterized by mutations in defined genes. APDS is classified into two subtypes: APDS1, caused by gain-of-function variants in the *PIK3CD* gene, and APDS2, associated with loss-of-function variants in the *PIK3R1* gene (1). These genetic alterations lead to constitutive activation of the PI3K-AKT-mTOR signaling pathway, contributing to lymphoproliferative manifestations and combined immunodeficiency (2, 3). APDS1 specifically involves pathogenic variants in the PI3Kδ catalytic subunit encoded by *PIK3CD*. These variants promote enhanced PI3Kδ activity, elevating phosphatidylinositol (3,4,5)-trisphosphate (PIP3) levels and facilitating downstream AKT activation in lymphocytes (4). This signaling dysregulation is associated with functional deficits in T and B cell populations, correlating with compromised antibacterial immunity. To date, whole-exome sequencing studies have identified 11 recurrent *PIK3CD* variants linked to APDS1 (*E1021K, E1025G, R929C, E81K, G124D, E525K, E525A, Y524N, N334K, R405C, C416R*) (1, 3, 5–11). with the E1021K substitution (c.3061G>A) accounting for approximately 85% of documented cases (12). APDS demonstrates notable clinical heterogeneity, spanning from asymptomatic presentations to severe combined immunodeficiency. The clinical spectrum typically includes enhanced susceptibility to bacterial and viral pathogens, prominent lymphoproliferative manifestations, and predisposition to malignancy development. Autoimmune phenomena may coexist in a subset of cases (4, 10, 13). Recurrent infections represent the predominant clinical presentation, with the majority of patients developing early-onset and severe respiratory tract infections including sinusitis, pharyngitis, tonsillitis, pneumonia, and empyema (14). Persistent herpes viral infections are particularly noteworthy, with Epstein-Barr virus (EBV) (28%) and cytomegalovirus (CMV) (14.7%) infections being frequently reported in case series (15). Approximately three-quarters of APDS1 patients develop benign lymphoproliferation, manifesting as chronic/recurrent generalized lymphadenopathy accompanied by hepatosplenomegaly. Concurrent gastrointestinal mucosal lymphoid hyperplasia commonly leads to chronic diarrhea and/or hematochezia (13, 16). Patients with APDS commonly develop autoimmune complications emerging after the first decade of life, which typically manifest as cytopenia and glomerulonephritis (17, 18). The immunological profile of APDS is characterized by combined T- and B-cell lymphopenia, hypogammaglobulinemia affecting IgA with concomitant normal or elevated IgM levels, and selective IgG subclass deficiencies (19, 20). In this report, we describe a genetically confirmed APDS1 patient presenting with EBV-positive diffuse large B cell lymphoma (EBV+ DLBCL), highlighting this rare complication in the setting of congenital immunodeficiency.

## Case presentation

A 23-year-old male presented with a six-year history of stable mild (≤1 cm) cervical lymphadenopathy first noted in 2015. Progressive nodal enlargement (1–2 cm) in December, 2021 prompted referral to our clinic. The patient’s medical history revealed childhood-onset bronchiectasis complicated by recurrent sinopulmonary infections, suggestive of underlying immunodeficiency.

**Figure 1:**
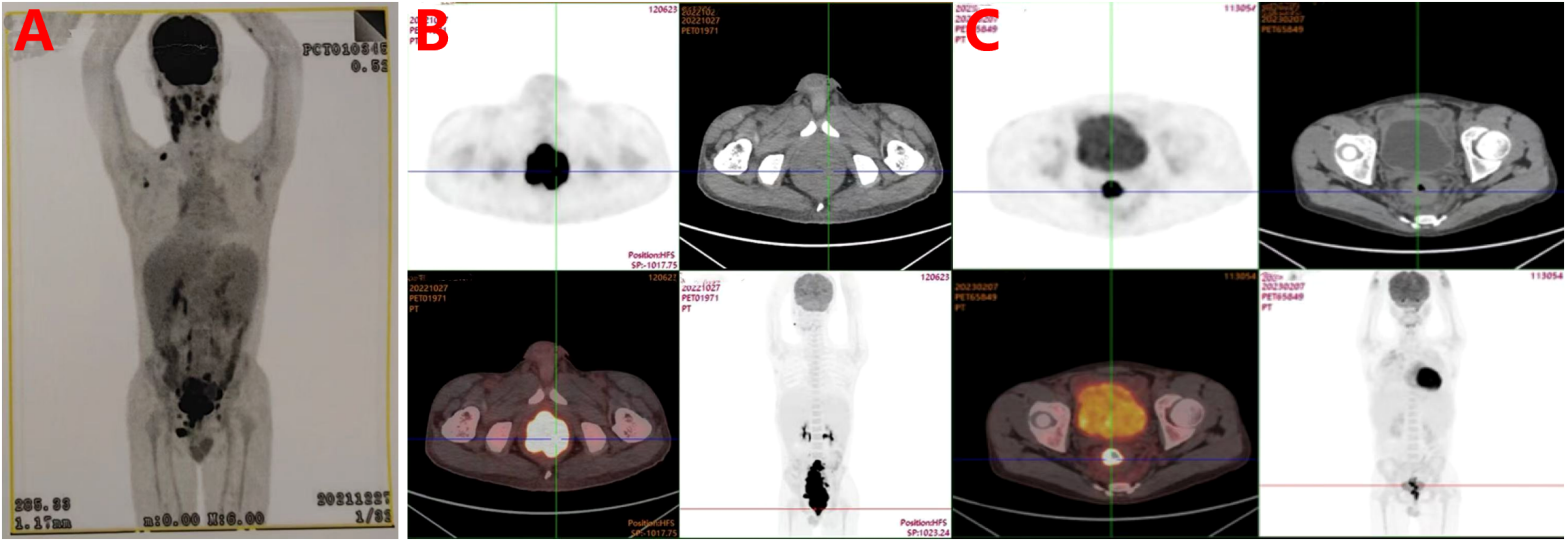
**(A)** Baseline whole-body PET-CT in December, 2021 revealed multistation hypermetabolic lymphadenopathy and rectal/ileocecal hypermetabolic lesions suggestive of lymphoma, with hepatosplenomegaly indicating reactive hyperplasia. Delayed rectal SUV retention and mild ileocecal tracer activity warrant clinicopathological correlation. **(B)** Diagnostic PET-CT in September, 2022 demonstrated a hypermetabolic sigmoidorectal mass (SUVmax=27.2) with disseminated lymphadenopathy meeting lymphoma criteria, accompanied by hepatosplenomegaly, sinusitis, and right lung collapse/bronchiectasis (our institution). **(C)** Interim PET-CT after four cycles of R-CHOPE demonstrated partial response with residual sigmoidorectal hypermetabolism (Deauville 5) and reduced nodal burden (size/number), though persistent nodal metabolic activity (Deauville 4) indicates incomplete remission.

The initial physical examination revealed marked craniofacial abnormalities, including mandibular prognathism with anterior crossbite and a disproportional facial structure, combined with concurrent underdevelopment of the vocal cords. Additionally, mobile submandibular lymphadenopathy was palpable bilaterally (1–2 cm in size), and splenomegaly was present, extending 4 cm below the left costal margin. Bilateral pulmonary crackles were also detected. Laboratory investigations revealed leukopenia (2.3×10^9^/L), hypogammaglobulinemia (IgA: 0.27 g/L), and inverted CD4+/CD8+ ratio (0.20). Elevated β2-microglobulin (3.5 mg/L) and detectable whole blood EBV-DNA (6.62×10² copies/mL) were noted, though plasma EBV load remained negative. PET/CT imaging revealed multifocal hypermetabolic lymphadenopathy with SUVmax values ranging from 5.8 to 12.4, involving the cervical, axillary, retroperitoneal, and iliac lymph node stations. Concurrent findings included splenomegaly (16.3 cm) and consolidation in the right middle lobe. (**Figure 1A**).

Clinical progression occurred in September 2022 with development of daily high-volume diarrhea (10×/day), abdominal pain, and respiratory symptoms including productive cough with purulent sputum. Emergent evaluation revealed cachexia with characteristic facial dysmorphism (low nasal bridge, mandibular prognathism with anterior crossbite, a disproportional facial structure), persistent lymphadenopathy (largest 2×2 cm right cervical node), and left lower quadrant tenderness, Anthropometric parameters confirmed growth retardation (height 168 cm; weight 63.2 kg). Laboratory studies confirmed iron-deficiency anemia (positive fecal occult blood) with concurrent hypoalbuminemia and hypocalcemia. Marked inflammatory activation was evidenced by elevated CRP (65.52 mg/L), IL-6 (36.13 pg/ml), and procalcitonin (0.08 ng/ml). Lymphocyte subset profiling demonstrated a marked predominance of CD3+CD8+ suppressor/cytotoxic T cells (87.30%) alongside depleted CD3-CD19+ B cell populations (3.10%), with corresponding Th/Ts ratio elevation to 69.60%. The analysis further revealed diminished CD3+CD4+ helper/inducer T cell proportions (7.40%) coexisting with attenuated CD3-CD16+CD56+ NK cell representation (0.20%), while CD3+ T cells constituted 13.70% of the total lymphocyte pool, including a 3.1% subset of CD4+CD25+CD127- regulatory T cells.

Serial imaging revealed progressive pulmonary infiltrates with right middle lobe atelectasis, radiologically consistent with infectious etiology. In contrast, the newly identified colorectal wall thickening demonstrating intense FDG avidity (SUVmax 8.6) was characterized as suspicious for metastatic lymphoma involvement (**Figure 1B**). Histopathological evaluation of rectal biopsies revealed diffuse infiltration by atypical large lymphoid cells characterized by vesicular chromatin, prominent nucleoli, and frequent mitoses (Ki-67 index >70%). Immunophenotypic analysis demonstrated positivity for B-cell markers (CD20, CD79a, PAX-5).

**Figure 2:**
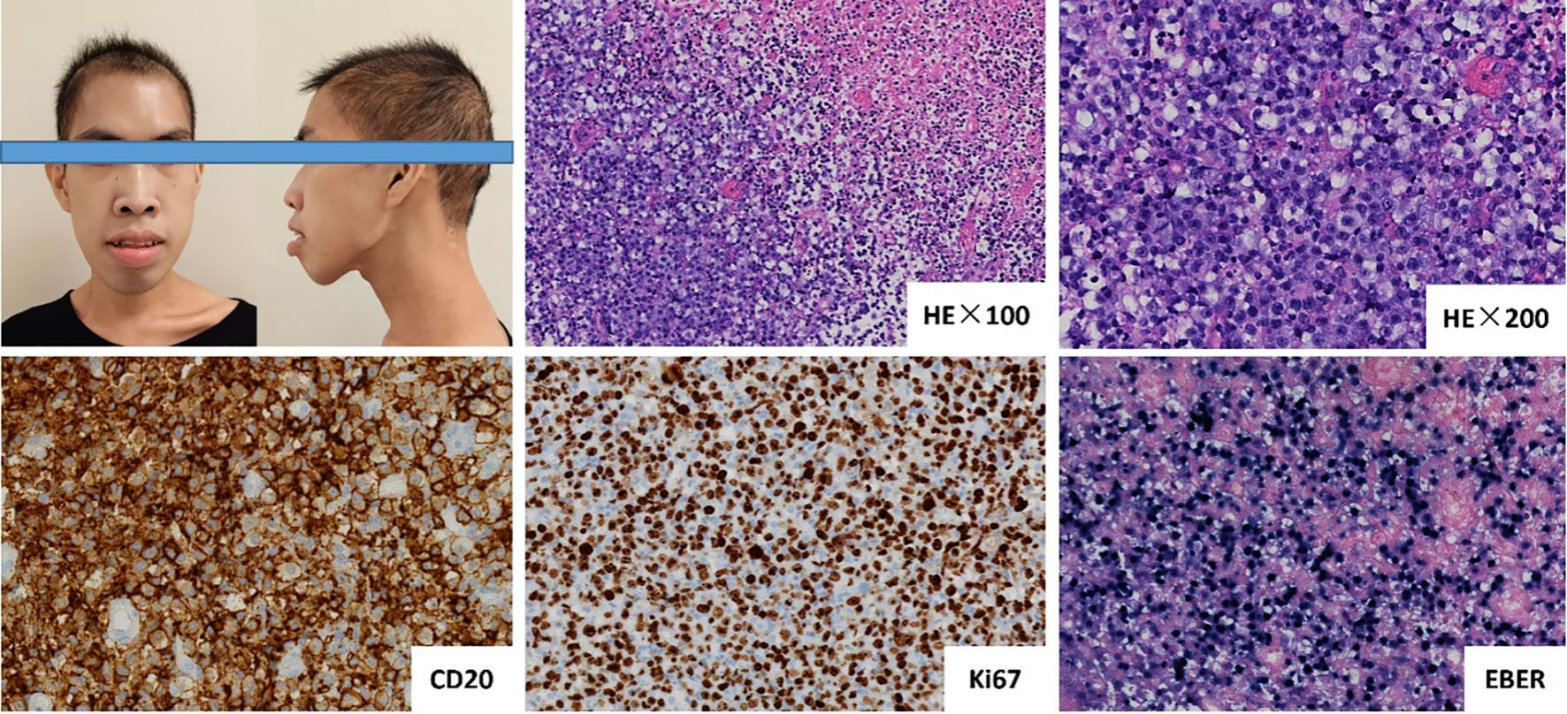
The patient exhibited distinct facial characteristics, including prominent mandible, and disharmonious facial proportions. Histopathological examination of rectal biopsies revealed diffuse infiltration by atypical large lymphoid cells, characterized by vesicular chromatin, prominent nucleoli, and frequent mitoses (Ki-67 index >70%). Immunophenotypic analysis demonstrated positivity for CD20, and Epstein-Barr virus (EBV) association was confirmed by EBER *in situ* hybridization.

EBV association was confirmed by EBER *in situ* hybridization (**Figure 2**). These findings established the diagnosis of EBV+ DLBCL. Whole-exome sequencing identified a pathogenic variant in the *PIK3CD* gene (c.3061G>A, p.Glu1021Lys), establishing a molecular diagnosis of APDS1. The treatment protocol commenced with dexamethasone bridging followed by a CHOP regimen (cyclophosphamide, doxorubicin, vincristine, prednisone) combined with etoposide, transitioning to rituximab-enhanced etoposide-modified CHOP (R-CHOPE: rituximab, etoposide, cyclophosphamide, pirarubicin, vindesine, dexamethasone) at cycle 2, which achieved transient EBV-DNA clearance (<4×10² copies/mL) in whole blood. Interim Positron Emission Tomography-Computed Tomography (PET-CT) at cycle 4 demonstrated partial metabolic response (Deauville 5-point scale score 3; **Figure 1C**). After the completion of 4 cycles of R-Chop chemotherapy, treatment was suspended for one cycle due to SARS-COV-2 infection. During this interim period, antiviral treatment was administered with Nirmatrelvir/Ritonavir (150 mg/100 mg twice daily). Considering the treatment-related myelosuppression following multi-agent chemotherapy, the dosing regimen of the PI3Kδ inhibitor was adjusted to Duvelisib (25 mg every 12 hours, for 14 days per month).

Augmented R-CHOPE therapy incorporating programmed death-1 (PD-1) inhibitor (sintilimab, 200 mg every 21 days) from cycle 5 ultimately induced complete metabolic remission by cycle 8 after a one-month treatment interval, Post-treatment evaluation following duvelisib-combined chemotherapy demonstrated progressive nodal regression to near-normal dimensions on serial imaging, with concurrent sintilimab maintenance therapy exhibiting a favorable safety profile devoid of clinically significant adverse events. Post-chemotherapy lymphocyte subset analysis revealed complete B-cell depletion (CD3-CD19 + 0.0%), accompanied by a Th/Ts ratio of 0.67 (CD3+CD4 + 35.30%; CD3+CD8 + 52.90%), elevated CD3+ T-cell predominance (91.9%), CD3-CD16+CD56+ NK cells at 6.6%, and regulatory T-cell subsets (CD4+CD25+CD127-) accounting for 5.8%. Longitudinal imaging surveillance identified persistent lymphadenopathy and splenomegaly; however, PET-CT revealed no hypermetabolic activity, radiologically consistent with effective suppression of residual lymphoma lesions following therapeutic intervention. The patient declined allogeneic hematopoietic stem cell transplantation (allo-HSCT) and is currently under close surveillance, remaining in complete remission (CR) without EBV reactivation for over one year.

## Literature review

We report a male adolescent with APDS1 presenting with lymphadenopathy, hepatosplenomegaly, bronchiectasis complicated by recurrent pulmonary infections and severe gastrointestinal symptoms. Whole-exome sequencing revealed a pathogenic *PIK3CD* variant (E1021K, c.3061G>A). Although parental genetic testing was unavailable, the clinical presentation strongly supports congenital pathogenesis. The immunophenotype demonstrated helper T-cell and B-cell lymphopenia with hypogammaglobulinemia (reduced IgA), contrasting with classic APDS features of hyper-IgM, IgG and IgM levels remained within normal ranges. Notably, the patient developed severe gastrointestinal manifestations, with histologically confirmed EBV+ DLBCL identified via rectal biopsy. This case illustrates an uncommon presentation of gastrointestinal EBV-positive DLBCL in APDS1. The co-occurrence of this lymphoproliferative complication with characteristic immune dysregulation emphasizes the diagnostic challenges associated with APDS phenotypic variability, particularly when accompanied by atypical immunological features.

Regarding clinical distinctions between subtypes, studies indicate a significantly higher prevalence of autoimmune comorbidities in APDS2 compared to APDS1 (12, 42). APDS2 is more characteristically associated with a phenotypic triad comprising growth retardation, mild cognitive impairment, and facial dysmorphism (e.g., hypertelorism, depressed nasal bridge, retrognathia) (43, 44). Notably, our APDS1 patient manifested overlapping features classically linked to APDS2, including craniofacial abnormalities (depressed nasal bridge, mandibular prognathism with anterior crossbite, facial disproportionality), laryngeal hypoplasia, and growth delay. Although no intellectual disability was clinically apparent, the lack of formal neuropsychological evaluation introduces uncertainty regarding subtle cognitive deficits, highlighting the importance of comprehensive phenotyping in APDS diagnostics.

EBV, an oncogenic gammaherpes virus with near-ubiquitous distribution, demonstrates seroprevalence exceeding 90% in adult populations worldwide (21). In immunocompetent hosts, primary infection typically occurs asymptomatically during childhood or manifests as self-limiting infectious mononucleosis (IM) in adolescents, classically presenting with fever, pharyngitis, and lymphadenopathy. Following initial exposure, EBV establishes lifelong latency through sophisticated immune evasion strategies. However, immunocompromised individuals or those with genetic predispositions may develop persistent IM-like symptoms accompanied by sustained viremia (>10^4^ EBV DNA copies/mL in peripheral blood), a clinical entity formally designated chronic active EBV infection (CAEBV) (22, 23). This case report delineates an atypical disease progression initially presenting with isolated lymphadenopathy that evolved into a complex syndrome featuring recurrent fever (>38.5°C), unintentional weight loss (>10% body mass index), multiple gastrointestinal ulcerations, and persistent high-grade viremia (EBV DNA load >10^5^ copies/mL). The clinical trajectory ultimately culminated in EBV-driven lymphomagenesis, specifically DLBCL confirmed through histopathological examination.

The oncogenic potential of EBV stems from its complex interplay with host cell machinery. Post-infection, the virus establishes latency primarily in memory B-cells, retaining capacity for periodic reactivation through latent-lytic cycle switching (24). Central to its transforming capacity, the EBV-encoded latent membrane protein 1 (LMP-1) constitutively activates NF-κB and STAT3 signaling pathways through structural mimicry of activated CD40 receptors, thereby driving malignant B-cell proliferation and clonal expansion (25–27). Concurrently, EBV employs multiple immune evasion tactics including downregulation of MHC class I/II molecules and induction of PD-L1 overexpression on infected cells, effectively impairing T-cell recognition and promoting T-cell exhaustion (28). These mechanisms synergistically create an immunosuppressive tumor microenvironment further augmented by viral induction of pro-inflammatory cytokines (29).

First characterized as age-related EBV-associated B-cell lymphoproliferative disorder (aEBV-LPD) by Oyama et al. (2003) (30), EBV-positive DLBCL gained formal recognition in the 2008 WHO classification as a distinct clinicopathological entity. While predominantly affecting immunocompetent adults over 50 years, emerging evidence documents sporadic cases in younger demographics (31). Although current standard-of-care R-CHOP immunochemotherapy achieves 60% 10-year disease-free survival for DLBCL (32), recent studies have revealed significantly poorer survival outcomes in EBV+ DLBCL patients compared to their EBV-negative counterparts (33), highlighting the distinct biological behavior of this lymphoma subtype.

The treatment strategies for APDS encompass non-specific therapies, targeted therapies, and allo-HSCT. Non-specific therapies include long-term anti-infection treatment combined with IVIG supplementation and inhibition of the PI3K-AKT-mTOR pathway, such as corticosteroids, azathioprine, mycophenolate mofetil, and cyclosporine. Targeted therapies for APDS focus on inhibiting the PI3K-AKT-mTOR pathway, with mTOR inhibitors (e.g., rapamycin/sirolimus) and selective PI3Kδ inhibitors (e.g., idelalisib or duvelisib) being the primary options, Recent studies have highlighted the potential curative role of allo-HSCT in APDS. A retrospective international study reported 2-year overall survival (OS) and failure-free survival rates of 86% and 68%, respectively (34). Additional studies demonstrated that APDS1 patients undergoing allo-HSCT achieved comparable survival rates without severe graft-versus-host disease (GVHD) and showed significant improvement in humoral immunity (35, 36). However, the application of allo-HSCT is limited due to the lack of long-term follow-up data and the risk of significant complications and high mortality. In the case presented, although the patient achieved a brief CR after eight cycles of R-CHOPE chemotherapy, the underlying congenital immunodeficiency poses a risk of relapse. The use of the PI3K inhibitor duvelisib may partially suppress PI3K pathway activation and improve immunodeficiency, as evidenced by the patient’s disease-free survival without EBV reactivation. However, allo-HSCT remains the only definitive approach to reconstituting the immune system and achieving a cure for this disease.

Given the limited documentation on secondary malignancies associated with APDS, this study conducted a systematic literature search across multiple databases including PubMed, Web of Science, and CNKI (China National Knowledge Infrastructure). The search strategy employed the following keywords: “APDS”, “PIK3CD hyperactivation syndrome”, and “PIK3CD”. Through comprehensive screening of relevant articles, we extracted and tabulated critical data elements encompassing APDS subtypes, tumor phenotypes, therapeutic interventions, and hematopoietic stem cell transplantation status (**Table 1**).

**Table 1:** Summary of case reports on secondary malignancies associated with APDS.

APDS subtypes	Histopathological tumor classifications (n = 24)	Age(years)	Sex	Infection type	Therapeutic interventions	Transplantation status	Prognosis	Year
APDS1	HL (n=1)	2.5	Male	EBV+	chemotherapy (COPP-ABVD)	allo-HSCT	alive	2020 (37)
APDS	PBL (n=1)	5	Female	EBV+	chemotherapy (CHOP) +Bortezomib	allo-HSCT	alive	2021 (38)
APDS2	interfollicular Hodgkin-like hyperplasia (n=1)	30	Female	CMV+ EBV+	ABVD +rapamycin	allo -HSCT	alive	2021 (39)
APDS1	BL (n=1)	25	Male	EBV+	chemotherapy (R-HyperCVAD )	ASCT +allo-HSCT	alive	2019 (40)
APDS1	germ cell tumor (n=1)	7	Female	EBV-	PEB+ Siromus	NO	alive	2023 (41)
APDS1	DLBCL (n=1)	21	Female	EBV-CMV-	chemotherapy (R-CHOP)	NO	alive	2014 (20)
APDS1	NHL (n=1)	36	Female	EBV-CMV-	chemotherapy (CHOP )	NO	alive	
APDS1	MZL (n=1)	24	Male	EBV+	NA	NA	NA	
APDS1/APDS2	DLBCL (n=7)	NA	NA	EBV+ (n=5) EBV- (n=2)	NA	HSCT	NA	2022 (34)
	HL(n =6)	NA	NA	EBV+( n=2) EBV-( n=2) NA(n=2)	NA	HSCT	NA	
	MZL(n =1)	NA	NA	EBV+( n=1) EBV-( n=1)	NA	HSCT	NA	
	MM(n =1)	NA	NA	NA	NA	HSCT	NA	
	T-cell lymphoma(n =1)	NA	NA	NA	NA	HSCT	NA	

The study cohort comprised 23 malignancies including Hodgkin lymphoma (HL, n=7), diffuse large B-cell lymphoma (DLBCL, n=8), Burkitt lymphoma (BL, n=1), plasmablastic lymphoma (PBL, n=1), non-Hodgkin lymphoma (NHL, n=1), marginal zone lymphoma (MZL, n=2), multiple myeloma (MM, n=1), T-cell lymphoma (n=1), and germ cell tumor (n=1). Pooled analysis demonstrated EBV infection in 13/23 cases, including a subset with CMV coinfection. CR was achieved through allo-HSCT in virally coinfected patients, while chemotherapy alone proved effective for those without viral involvement. Notably, one case of interfollicular Hodgkin-like hyperplasia (n=1) was identified in a patient with inborn errors of immunity (IEI) (45). Initially misdiagnosed as HL, the diagnosis was revised following comprehensive evaluation. This patient achieved sustained remission through combined chemotherapy, rapamycin, and allo-HSCT, remaining alive at last follow-up.

These findings suggest that viral coinfections may amplify immune dysregulation in APDS. The diagnostic complexity is further compounded by lymphoproliferative manifestations in IEI, where lymphoma-mimicking lymphadenopathy creates significant challenges in differentiating benign hyperproliferation from hematologic malignancies. Notably, the observed therapeutic synergy between PI3Kδ inhibitors and chemotherapy demonstrates potential for reducing transplantation dependency, especially in clinically complex APDS presentations.

Notably, the largest cohort in this analysis derived from an international multicenter retrospective study involving 57 APDS patients, among whom 13 developed secondary malignancies. While this study primarily evaluated outcomes of allo-HSCT in APDS, it did not separately analyze prognosis in those with hematologic malignancies. However, the median follow-up of 26.3 months and a 2-year overall survival (OS) rate of 86% among transplanted patients underscore allo-HSCT as a viable strategy for prolonging survival. Furthermore, one case highlighted a patient with Burkitt lymphoma who relapsed after autologous stem cell transplantation (ASCT); subsequent genetic confirmation of APDS prompted salvage chemotherapy followed by allo-HSCT, achieving sustained CR. The sole germ cell tumor case achieved CR through surgical resection combined with immunotherapy, emphasizing the efficacy of multimodal approaches.

APDS is pathologically characterized by aberrant lymphocyte proliferation and heightened oncogenic predisposition. Mechanistic investigations reveal that PI3K signaling dysregulation drives tumorigenesis through pathological accumulation of phosphatidylinositol-3,4,5-trisphosphate (PtdInsP3) (46). This lipid secondary messenger activates the AKT/PKB-mTOR signaling axis, thereby mediating malignant phenotypes including uncontrolled proliferation, apoptosis resistance, metabolic reprogramming, and enhanced cellular migration (47). Notably, this constitutive pathway activation stems from functional loss of PTEN, a tumor suppressor that normally dephosphorylates PtdInsP3. Genetic mutations or epigenetic silencing of PTEN establishes a self-reinforcing oncogenic loop through sustained PI3K signaling (48, 49). Lineage-specific mechanistic analyses demonstrate distinct pathogenic consequences of PI3K hyperactivation: In T lymphocytes, aberrant PI3K signaling drives pathological expansion and survival programs, precipitating autoimmune manifestations and T-cell leukemogenesis (49). Conversely, B-cell PI3K dysregulation disrupts normal differentiation checkpoints while promoting proliferative signaling, thereby accelerating B-cell lymphoma progression (50, 51).

At the level of therapeutic intervention, selective PI3Kδ inhibitors (e.g., leniolisib) primarily modulate the AKT/PKB-mTOR signaling axis through competitive binding to the ATP-binding domain of the p110δ catalytic subunit. This mechanism effectively suppresses kinase activity and reduces pathological PtdInsP3 accumulation. Clinical studies have revealed that the co-occurrence of immunodeficiency and malignancy in APDS patients suggests multiple immune evasion mechanisms: (1) defective tumor antigen presentation mechanisms leading to immune recognition escape; (2) immunosuppressive microenvironments impairing effector cell function; and (3) aberrant activation of anti-apoptotic pathways conferring resistance to immune-mediated cytotoxicity (52). These synergistic mechanisms enable malignant cells to evade immune surveillance and sustain persistent proliferation. Recent phase III clinical trials have demonstrated sustained clinical benefits and favorable safety profiles of isoform-specific PI3Kδ inhibitors (e.g., leniolisib) in APDS management (45). However, incomplete blockade of tumor escape pathways by these agents may limit therapeutic efficacy in certain cases. In this context, allo-HSCT demonstrates unique advantages through dual mechanisms of action - hematopoietic system reconstitution and immune surveillance restoration - significantly mitigating risks of tumor immune evasion.

This study systematically analyzes the disease spectrum characteristics of secondary malignancies in APDS, validating the clinical value of combined allogeneic HSCT and precision targeted therapy. Our comprehensive evaluation not only enhances understanding of APDS pathogenesis but also establishes a theoretical framework for evidence-based management strategies in this rare immunodeficiency disorder.

## Conclusion

APDS, an inborn error of immunity, is strongly associated with EBV-mediated lymphomagenesis. Here, we describe a rare case of APDS complicated by EBV-positive DLBCL. Current APDS management strategies involve antimicrobial prophylaxis, immunoglobulin supplementation, immunosuppressive regimens, PI3K inhibitors, and allo-HSCT. Although chemotherapy may induce partial remission in conventional DLBCL, immunodeficiency-associated DLBCL often demonstrates limited therapeutic responsiveness to standard treatment. In patients with concurrent inborn error of immunity and malignancy, allo-HSCT offers dual benefits: restoration of functional immunity and direct tumor suppression, potentially achieving complete remission. These observations suggest allo-HSCT may represent the sole potentially curative intervention for APDS patients with malignancy, particularly when conventional therapies fail to address the underlying immune dysfunction.

## Data Availability

The original contributions presented in the study are included in the article/supplementary material. Further inquiries can be directed to the corresponding author/s.
