# Correction: EBV-positive diffuse large B cell lymphoma secondary to activated phosphoinositide 3 kinase δ syndrome type 1 (APDS1): a case report and literature review

**DOI:** 10.3389/fimmu.2025.1659197

**Published:** 2025-07-15

**Authors:** Qiu-yuan Xiang, Min Zuo, Ji-Hao Zhou, Chun Feng

**Affiliations:** ^1^ Department of Hematology, The Second Clinical Medical College of Jinan University, Shenzhen, China; ^2^ Department of Pathology, Shenzhen People’s Hospital (The Second Clinical Medical College of Jinan University; The First Affiliated Hospital of Southern University of Science and Technology), Shenzhen, China; ^3^ Department of Hematology, Shenzhen People’s Hospital (The Second Clinical Medical College of Jinan University; The First Affiliated Hospital of Southern University of Science and Technology), Shenzhen, China

**Keywords:** activated phosphoinositide 3-kinase δ syndrome, Epstein-Barr virus, diffuse large B-cell lymphoma, Epstein-Barr virus-positive diffuse large B-cell lymphoma (EBV+ DLBCL), inborn error of immunity

Wrong affiliation, but not replacing with present address

Author “Min Zuo” was erroneously assigned to affiliation “Department of Pathology, The Second Clinical Medical College of Jinan University, Shenzhen, China”.

The correct affiliation is “Department of Pathology, Shenzhen People’s Hospital (The Second Clinical Medical College of Jinan University; The First Affiliated Hospital of Southern University of Science and Technology), Shenzhen, China”.

Authors “Ji-Hao Zhou and Chun Feng” were erroneously assigned to affiliation “Department of Hematology, The Second Clinical Medical College of Jinan University, Shenzhen, China”.

The correct affiliation is “Department of Hematology, Shenzhen People’s Hospital (The Second Clinical Medical College of Jinan University; The First Affiliated Hospital of Southern University of Science and Technology), Shenzhen, China”.

The original version of this article has been updated.

